# Supplementary material for: Synthesis and Biological Evaluation of Thiazole-Based Derivatives with Potential against Breast Cancer and Antimicrobial Agents
Source: Int J Mol Sci. 2022 Aug 30;23(17):9844. doi: 10.3390/ijms23179844 (PMC9456159; doi:10.3390/ijms23179844)
Supplement: Supplementary file 1 [file ijms-23-09844-s001.zip › ijms-1865810-supplementary/Supplementary Materials.pdf]

Supplementary Materials

Synthesis and Biological Evaluation of Thiazole-Based Derivatives as Potential Anticancer and Antimicrobial agents

Ekaterina Pivovarova [1*], Alina Climova [1], Marcin Świątkowski [1], Marek Staszewki [2], Krzysztof Walczyński [2], Marek Dzięgielewski [2], Marta Bauer [3], Wojciech Kamysz [3], Anna Krześlak [4], Paweł Jóźwiak [4], Agnieszka Czylkowska [1*]

[1] Institute of General and Ecological Chemistry, Lodz University of Technology, Żeromskiego 114, Łódź, 90-543, Poland
[2] Department of Synthesis and Technology of Drugs, Medical University, Muszyńskiego Street 1, 90-145 Łódź, Poland
[3] Department of Inorganic Chemistry, Faculty of Pharmacy, Medical University of Gdańsk, 80-416 Gdańsk, Poland
[4] Department of Cytobiochemistry, Faculty of Biology and Environmental Protection, University of Lodz, 90-236 Łódź, Poland

* Correspondence: ekaterina.pivovarova@dokt.p.lodz.pl;agnieszka.czylkowska@p.lodz.pl.

NMR spectra of ligands L1-L3:

Figure S1. ^{1}H NMR spectrum of L1.

Figure S2. ^{13}C NMR spectrum of L1.

Figure S3. ^{1}H NMR spectrum of L2.

Figure S4. ^{13}C NMR spectrum of L2.

Figure S5. ^{1}H NMR spectrum of L3.

Figure S6. ^{13}C NMR spectrum of L3.

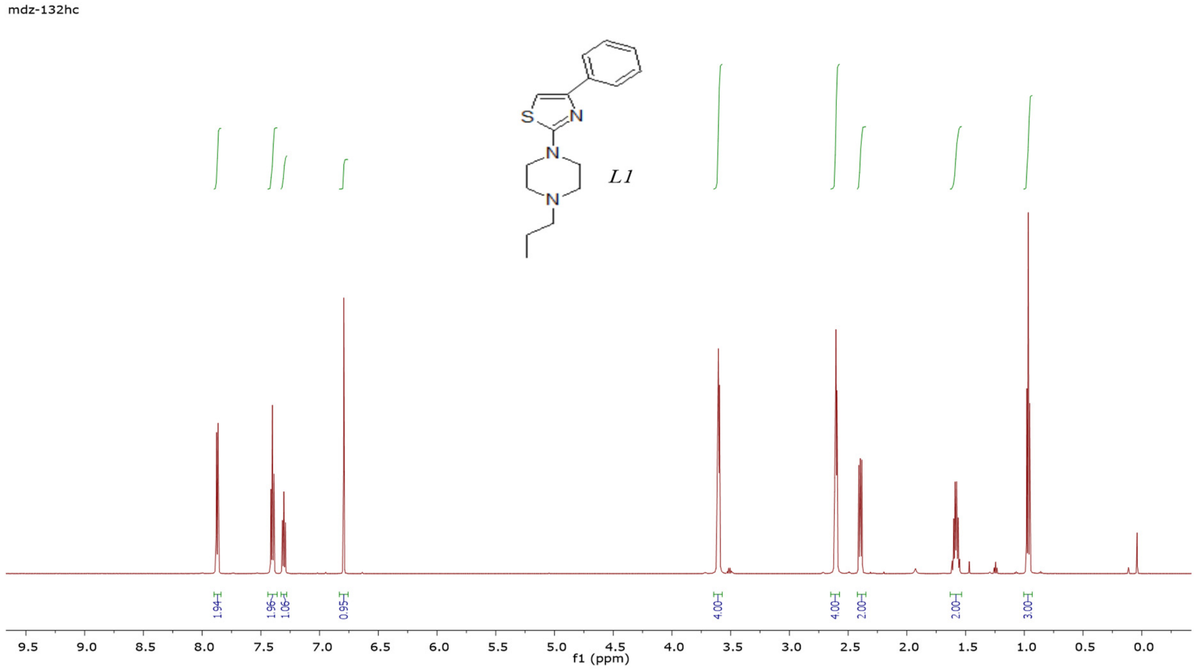

Figure S1. ^{1}H NMR spectrum of L1 (600 MHz, $CDCl_3$).

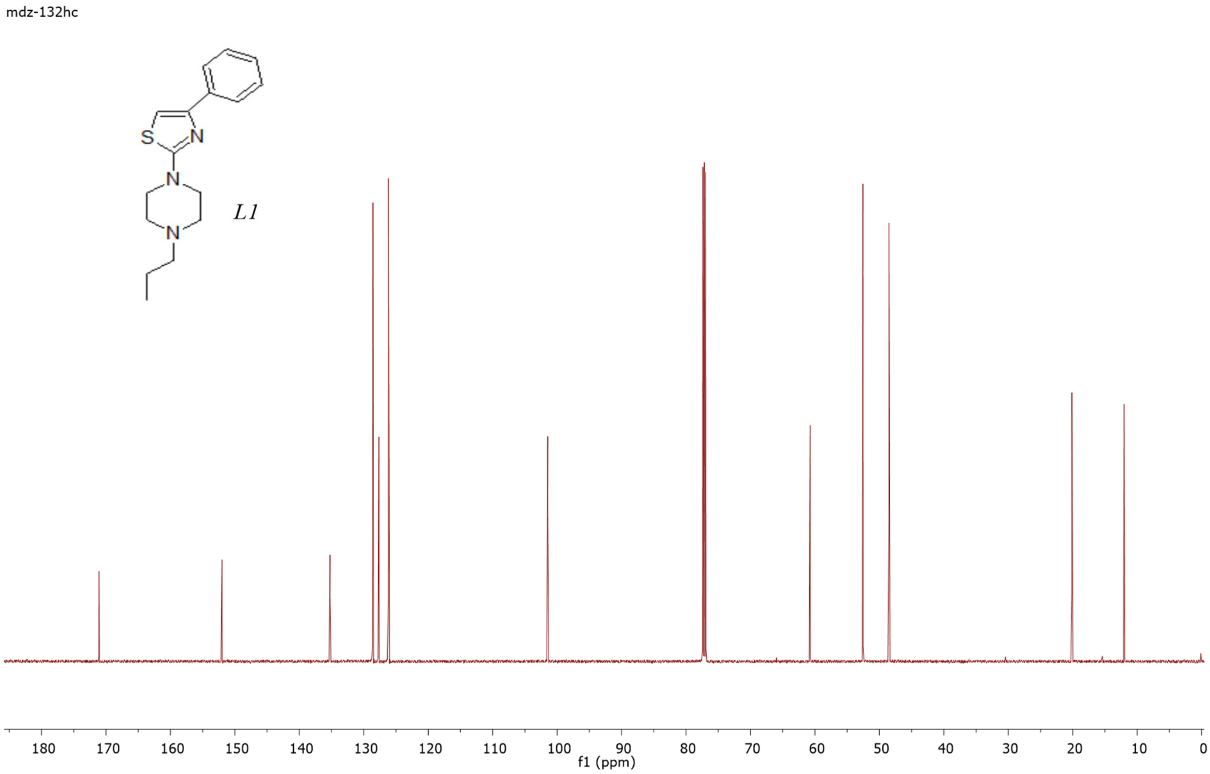

Figure S2. ^{13}C NMR spectrum of L1 (150 MHz, $CDCl_3$).

mdz-133

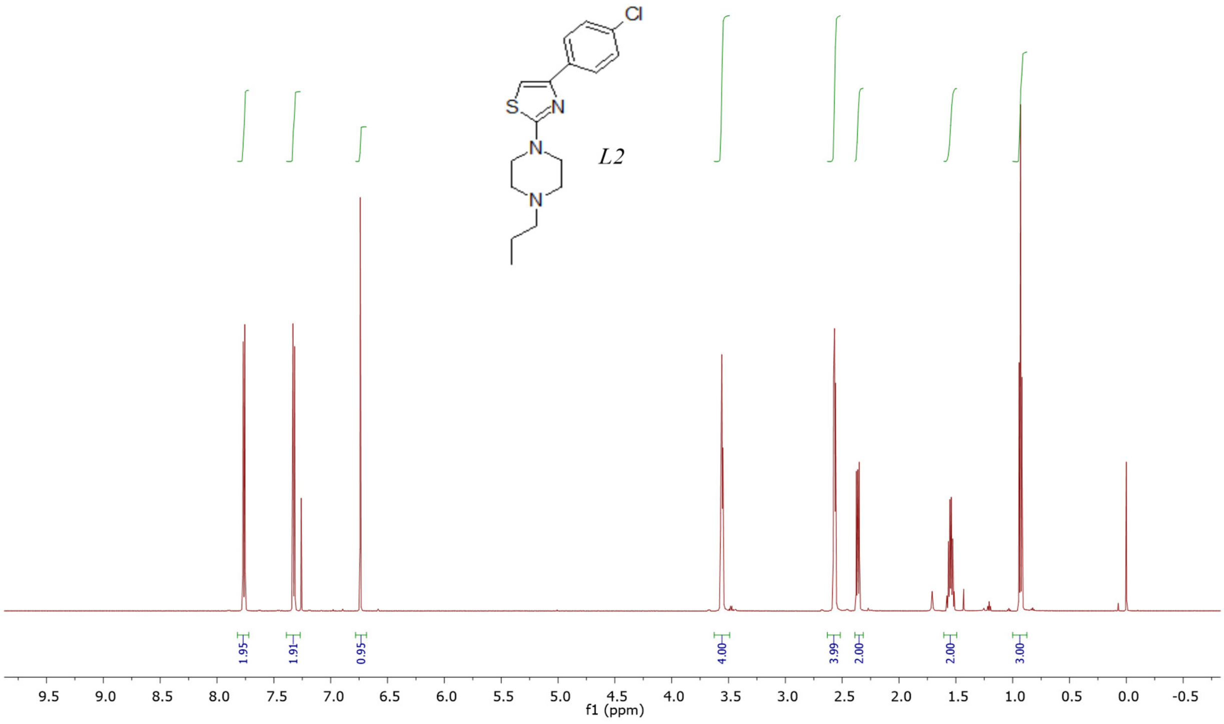

Figure S3. ^{1}H NMR spectrum of L2 (600 MHz, $CDCl_3$).

mdz-133c

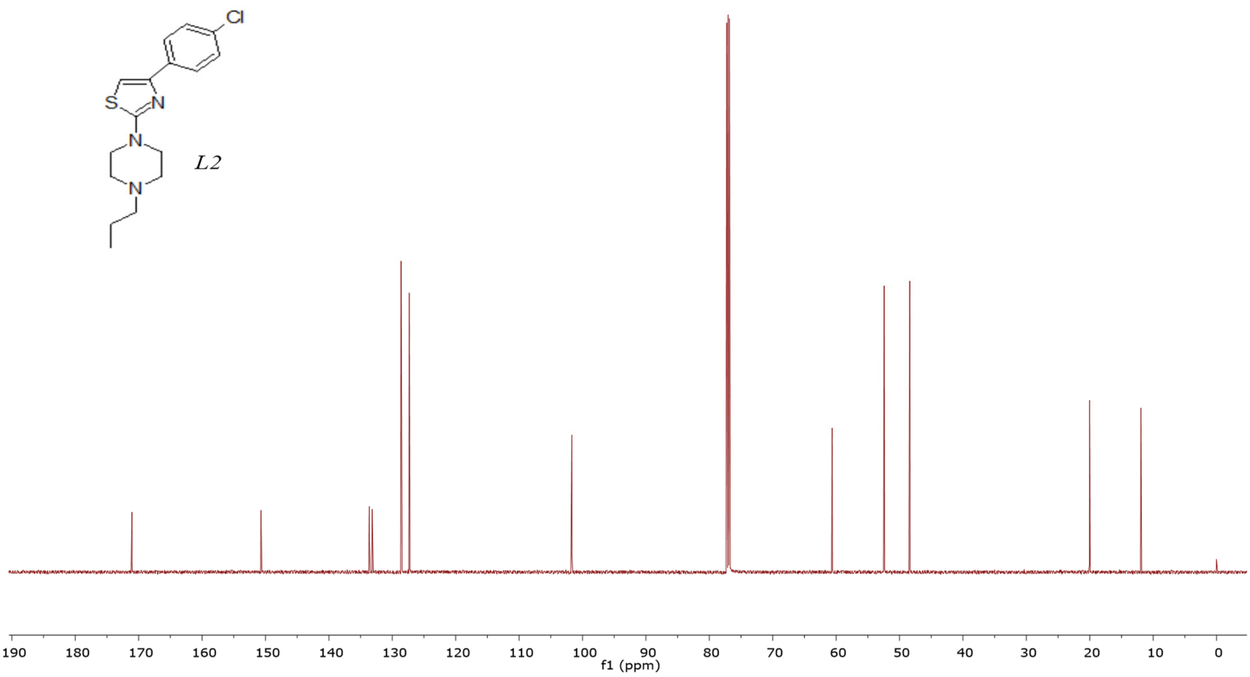

Figure S4. ^{13}C NMR spectrum of L2 (150 MHz, $CDCl_3$).

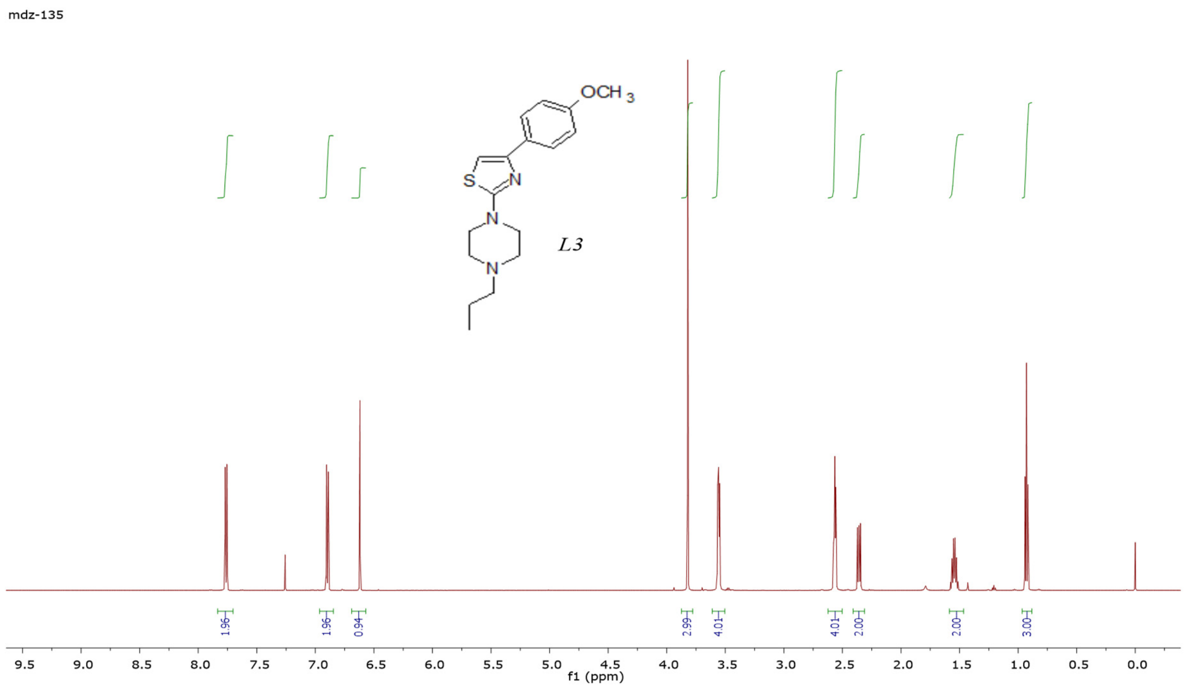

Figure S5. ^{1}H NMR spectrum of L3 (600 MHz, $CDCl_3$).

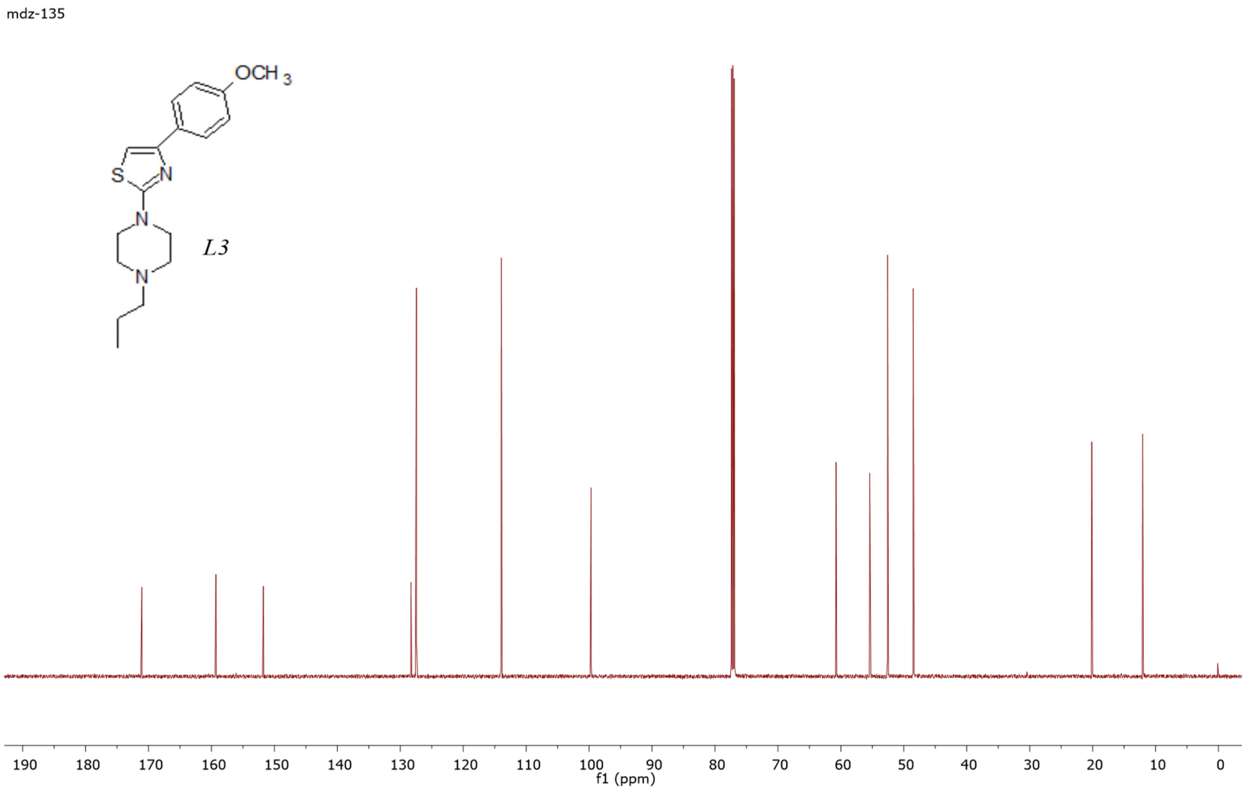

Figure S6. ^{13}C NMR spectrum of L3 (150 MHz, $CDCl_3$).
